# Detection of Drug-Induced Thrombocytopenia Signals in Children Using Routine Electronic Medical Records

**DOI:** 10.3389/fphar.2021.756207

**Published:** 2021-11-12

**Authors:** Xiaolu Nie, Lulu Jia, Xiaoxia Peng, Houyu Zhao, Yuncui Yu, Zhenping Chen, Liqiang Zhang, Xiaoling Cheng, Yaqi Lyu, Wang Cao, Xiaoling Wang, Xin Ni, Siyan Zhan

**Affiliations:** ^1^ Department of Epidemiology and Biostatistics, School of Public Health, Peking University, Beijing, China; ^2^ Center for Clinical Epidemiology and Evidence-based Medicine, Beijing Children’s Hospital, Capital Medical University, National Center for Children’s Health, Beijing, China; ^3^ Department of Pharmacy, Beijing Children’s Hospital, Capital Medical University, National Center for Children’s Health, Beijing, China; ^4^ Hematologic Disease Laboratory, Beijing Children’s Hospital, Capital Medical University, National Center for Children’s Health, Beijing, China; ^5^ Hematology Center, Beijing Children’s Hospital, Capital Medical University, National Center for Children’s Health, Beijing, China; ^6^ Department of Medical Record Management, Beijing Children’s Hospital, Capital Medical University, National Center for Children’s Health, Beijing, China; ^7^ Beijing Children’s Hospital, Capital Medical University, National Center for Children’s Health, Beijing, China; ^8^ Center for Intelligent Public Health, Institute for Artificial Intelligence, Peking University, Beijing, China

**Keywords:** drug-induced thrombocytopenia, signal detection, children, electronic medical records, post-marketing pharmacovigilance

## Abstract

**Background:** Drug-induced thrombocytopenia (DITP) is a severe adverse reaction and a significantly under-recognized clinical problem in children. However, for post-marketing pharmacovigilance purposes, detection of DITP signals is crucial. This study aimed to develop a signal detection model for DITP using the pediatric electronic medical records (EMR) data.

**Methods:** This study used the electronic medical records collected at Beijing Children’s Hospital between 2009 and 2020. A two-stage modeling method was developed to detect the signal of DITP. In the first stage, we calculated the crude incidence by mining cases of thrombocytopenia to select the potential suspected drugs. In the second stage, we constructed propensity score–matched retrospective cohorts of specific screened drugs from the first stage and estimated the odds ratio (OR) and 95% confidence interval (CI) using conditional logistic regression models. The novelty of the signal was assessed by current evidence.

**Results:** In the study, from a total of 839 drugs, 21 drugs were initially screened as potentially inducing thrombocytopenia. In total, we identified 18 positive DITP associations. Of these, potential DITP risk of nystatin (OR: 1.75, 95% CI: 1.37–2.22) and latamoxef sodium (OR: 1.61, 95% CI: 1.38–1.88) were two new DITP signals in both children and adults. Six associations between thrombocytopenia and drugs including imipenem (OR: 1.69, 95% CI: 1.16–2.45), teicoplanin (OR: 4.75, 95% CI: 3.33–6.78), fusidic acid (OR: 2.81, 95% CI: 2.06–3.86), ceftizoxime sodium (OR: 1.83, 95% CI: 1.36–2.45), ceftazidime (OR: 2.16, 95% CI: 1.58–2.95), and cefepime (OR: 5.06, 95% CI: 3.77–6.78) were considered as new signals in children.

**Conclusion:** This study developed a two-stage algorithm to detect safety signals of DITP and found eighteen positive signals of DITP, including six new signals in a pediatric population. This method is a promising tool for pharmacovigilance based on EMR data.

## Introduction

Drug-induced thrombocytopenia (DITP) is an adverse reaction and a significantly under-recognized clinical problem. The platelet count is usually less than 100×10^9^/L; therefore, DITP can often lead to abrupt and severe bleeding complications and even death (Vayne et al., 2020; Doodnauth et al., 2021). DITP deserves special attention since its cumulative incidence is 10 cases per million population per year, with a prevalence as high as 25% in critically ill patients (Danese et al., 2020). The worldwide incidence of DITP in hospitalized patients ranges from 2.26 to 4.99% (ten Berg et al., 2009; Seco-Melantuche et al., 2013). It has been reported that more than 300 medications, including antibiotics (Butt et al., 2019; Savage-Elliott et al., 2020), neurological drugs (Kim et al., 2020), and antineoplastic agents (Tam et al., 2019), could lead to DITP in the adult population. However, children have an immature organ function and a different spectrum of disease compared with adults. Thus, drugs may induce more severe adverse reactions in children, and the relative evidence from adults could not directly apply to the pediatric population. In addition, the evidence from clinical trials in the pediatric population is insufficient because of the difficult recruitment of participants and ethical considerations. Hence, accurate methods for post-marketing drug safety surveillance and signal detection of DITP in children are urgently needed (Reese et al., 2013).

A spontaneous reporting system (SRS) remains the cornerstone of post-marketing drug safety surveillance at present in China, despite its limitations, such as lack of denominator data to calculate incidence, underreporting, and delayed reporting of adverse drug reactions (ADRs). Considering the limitation of the passive surveillance system, active surveillance using routinely collected health data, such as electronic medical records (EMRs), has become an essential complementary method for drug safety in the clinical practice (Pacurariu et al., 2015; Yang et al., 2018). Compared to SRS, the longitudinal EMR database contains clinical data on patients, especially the prescribed off-label drugs in child patients with longer follow-ups. Therefore, it is advantageous to analyze EMR data to detect safety signals of drugs for real-time pharmacovigilance and evaluate the benefit/risk profile of newly approved and older drugs.

Several studies have been conducted to develop methods for detecting DITP signals using electronic health records (Moore et al., 2009; Ramirez et al., 2010; Lee et al., 2019) using laboratory test results and narrative texts. The findings showed that linezolid, ganciclovir, and 5-fluorouracil were potentially associated with thrombocytopenia. However, these studies mainly focused on adult patients, and, to date, little is known about children (Osokogu et al., 2016).

This study aimed to develop a two-stage procedure to detect signals of DITP in the child population using EMR data, and provide candidate drugs for further precise drug monitoring and causality validation studies.

## Materials and Methods

### Data Sources

We conducted this retrospective cohort study using Beijing Children’s Hospital (BCH) longitudinal inpatient database, which has been described previously (Wei et al., 2019). If a person with the same patient ID were hospitalized more than once, we identified them as different records. This study used inpatients’ data from 1 January 2009 to 31 December 2020. These data encompassed health information (including medical orders of doctors, diagnosis records from the Hospital Information System, and laboratory tests from the Laboratory Information System and drug prescriptions) on approximately 426,000 inpatients under 18 years of age.

The study was conducted in accordance with the Declaration of Helsinki. The protocol was approved by the Institutional Review Board (IRB) of Beijing Children’s Hospital, Capital Medical University (approval number: 2018–129), with a waiver of informed consent. All the data we used have been de-identified to protect patients’privacy and confidentiality. This study was reported according to the RECORD-PE statement.

### Study Population Identification

Eligible participants were patients aged 28 days to 18 years old who were administered in the BCH from January 2009 to December 2020. All participants had at least two laboratory test records of platelet count and drug prescriptions in the data warehouse. Considering the temporal relationship between drug and events, patients whose initial platelet count (PLT) was < 100×10^9^/L after study entry were excluded (Harinstein et al., 2012).

### Laboratory Criterion of DITP

The laboratories of BCH are certified and accredited under the appropriate International Organization for Standardization standards. According to the Guidelines for Medical Nomenclature Use of Adverse Drug Reactions, issued by the National Center for ADR Monitoring of the China Food and Drug Administration (CFDA) in 2016 and the method of IHI Global ADR Trigger Tool (Reese et al., 2013), the trigger of DITP in this study was defined as PLT lower than 100×10^9^/L after administration of a particular medicine within the appropriate therapeutic dose range.

### Screening Suspected Drugs Potentially Associated With DITP

**FIGURE 1:**
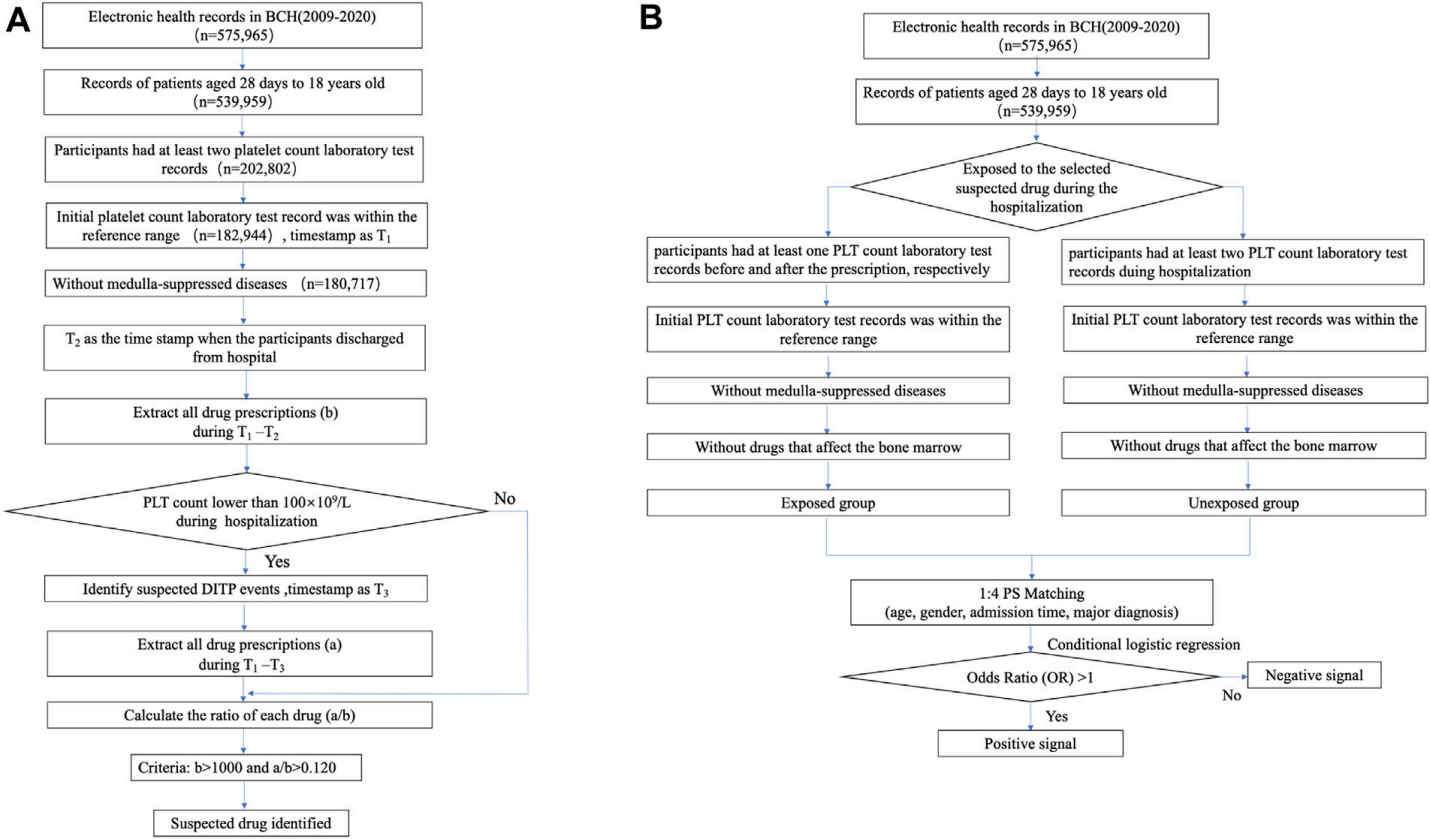
Workflow of two-step signal detection method of DITP using EMR data. **(A)** The workflow of stage one for screening drugs potentially causing DITP. **(B)** The overall design of stage two for the detection of DITP signals based on retrospective cohort design.

To identify suspected drugs potentially associated with DITP for further association analysis, we developed a fifth-step workflow (see in Figure 1A). Only non-chemotherapy drugs were involved in this study since chemotherapy agents have a myelosuppressive effect. All the involved drugs were unified with generic names and mapped with the Anatomical Therapeutic Chemical (ATC) code. When a patient used two or more drugs in one prescription record, we counted the number of users in each drug, respectively. Duplicate prescriptions of the same drug in each admission were counted only once. The main steps were as follows:

1) Considering the confounding by indication, we excluded the records of patients containing a diagnosis of diseases that affects PLT (shown in Supplementary Table S1). The remaining hospitalization records were defined as Group 1.
2) The time when a patient in Group 1 obtained an initial normal platelet count results after admission was signed as Timestamp 1 (T_1_), and the time for discharge of each hospitalization of every involved patient was labeled as Timestamp 2 (T_2_). We calculated the number of drug users (b) during the period of T_1_–T_2_.
3) The hospitalization records of patients in Group 1, which were potential DITP events during T_1_–T_2_ according to the definition of DITP trigger, were included in Group 2. We labeled the time of PLT level lower than 100×10^9^/L as Timestamp 3 (T_3_).
4) We calculated the number of users for each medicine in Group 2 who were identified by DITP trigger (a) during the period of T_1_–T_3_.
5) The ratio a/b for each drug was calculated. The suspected drug met the following criteria were selected for further association analysis: 1) the ratio a/b > 0.120, considering the a/b values of solvents for intravenous infusions, such as normal saline and glucose injection, ranged from 0.092 to 0.118, which can be regarded as the value of background since it is well known that normal saline and glucose injection have no effect on DITP; 2) number of total users (b) > 1,000, ensuring sufficient sample size and adequate power.

### Definition of Suspected Drug Cohorts

According to the above screening procedure of suspected drugs, we conducted a series of retrospective propensity score-matched cohort studies to detect the association between suspected drugs and DITP by comparing differences in DITP event rates between the exposed and unexposed groups.

#### Exposed Group

The eligible participants were required to be prescribed a specific screened drug after admission to BCH and had at least two PLT results before and after taking the specific suspected drug, respectively. The date of initial prescription of a specific drug was considered the index time for the corresponding participant. Patients had to have an initial result of platelet count ≥100×10^9^/L before index time. To accurately assess the drug–DITP associations, patients who were diagnosed with medulla-suppressed diseases (shown in Supplementary Table S1) or received prescriptions of the thrombocytopenic agents (shown in Supplementary Table S2) (Reese et al., 2010) before the first abnormal test of PLT were also excluded.

#### Unexposed Group

The patients without prescriptions of specific suspected drugs were initially selected to the unexposed group. Among them, we chose the participants with at least two platelet count tests from admission to discharge and had an initial result of platelet count ≥100×10^9^/L. For the same selection considerations as the exposure group, we excluded patients diagnosed with medulla-suppressed diseases or who had prescriptions of thrombocytopenic agents.

Each suspected drug of the screening stage was considered as exposure and was examined in a cohort study according to the above procedures. Follow-up ended until the first occurrence of the following events: platelet count <100×10^9^/L, discharged from BCH, or December 31, 2020. The overall main framework of this study is displayed in Figure 1B.

### Signal Detection and Novelty Assessment

The odds ratio (OR) and its 95% confidence interval (CI) were estimated to assess the association between specific suspected drugs and the incidence of DITP events. The signal of DITP was positive if the lower limit of the 95% CI of OR was greater than 1.0; otherwise, it was regarded as a negative signal.

Not all statistically significant associations could be regarded as potential new signals. Thus, further validation was needed to evaluate these signals. Since there was no recognized gold standard for evaluating the relevance of the DITP association, we performed a manual review of the summary of product characteristics (SPCs) included in the Micromedex, DXY Drugs Information, and electronic literature databases, including PubMed, Embase, and China National Knowledge Infrastructure and Wanfang Database. In addition, according to the published literature about adults and children, George JN et al. established DITP-related drug database, which had been updated till 2018 (George, 2015). According to the report status in SPCs, literature from electronic databases, and database of DITP-related drugs, we defined two types of new DITP signals for children: (I) The specific drug–DITP signal had never been reported in the summary of product characteristics or in the literature; (II) the specific drug signal had been reported in the literature about adults, but no reports about children could be found in the literature.

### Statistical Analysis

We compared the baseline characteristics of each screened suspected drug group and the unexposed group. For each patient, we calculated person-time of follow-up as the amount of time from the index time to the end of follow-up. DITP incidence rates were calculated for each cohort. We calculated propensity scores for the initial prescription of a specific suspected drug using the logistic regressions. The variables included in the model included age, gender, admission time, and major diagnosis (based on the classification in ICD-10). For a particular suspected drug, the records from the exposed group were matched 1:4 to those of the unexposed group using the caliper matching method (caliper equaled 0.1). Then we compared the OR of DITP in each specific suspected drug cohort with the corresponding unexposed group cohorts using conditional logistic regression models. Patients with missing values for age, gender, and admission date were excluded from the analysis. We also performed sensitivity analyses to assess the robustness of our findings. We used the propensity score regression method other than matching in the primary analysis.

All *p* values were two-sided, and *p* < 0 0.05 was considered significant for all tests. MySQL software version 14.14 (Oracle, California, United States) was used as the database management system to extract the required data from BCH’s EMR database. Data were processed and summarized using the pandas v1.2.2 model in *Python* 3.7. R 3.5.2 software (R Foundation for Statistical Computing, Vienna, Austria. ISBN 3-900051-00-3) was used for statistical analysis, and SAS 9.4 TS Level M5 (SAS Institute Inc. Cary, NC, United States) was used for forest plot demonstrating the results of association analysis.

## Results

### Selection of Suspected Drugs

After combining drugs with the same ingredients and ATC but different dosages and forms, 388 drugs remained. Among these drugs, 210 satisfied the screening criteria that the total number of drug users was >1,000, and the rate of a/b was beyond 0.12. After excluding the chemotherapy drugs and drugs that affected PLT count, 186 suspected drugs were identified as suspected drugs and were selected for further analysis for DITP signals. Among them, 21 drugs met the inclusion criteria (b > 1,000 and a/b > 0.12). These were amphotericin B, chlorpheniramine, vancomycin, imipenem, fluconazole, sulfamethoxazole, loratadine, meropenem, promethazine hydrochloride, teicoplanin, nystatin, fusidic acid, ceftizoxime sodium, ceftazidime, cefpiramide, cefepime, linezolid, cefoperazone sodium and sulbactam sodium, milrinone, heparin, and latamoxef sodium. These twenty-one drugs were considered as suspected drugs and chosen for DITP signals detection in stage 2 (Table 1).

**TABLE 1:** Suspected drugs associated with thrombocytopenia in the pediatric population.

Drug name	Pharmacological classification	ATC code	Number of DITP events(a)	Total number of usages(b)	Ratio (a/b)
Amphotericin	Antifungal drug	J02AA01	656	1,198	0.548
Chlorpheniramine	Antihistaminic	R06AB04	7,859	15,725	0.500
Vancomycin	Polypeptide antibiotic	J01XA01	6,636	13,886	0.478
Imipenem	Beta-lactam antibiotic	J01DH51	764	1,762	0.434
Fluconazole	Antifungal drug	J02AC01	2,797	8,295	0.337
Sulfamethoxazole	Sulfonamides and trimethoprim	J01EE01	7,477	23,550	0.317
Loratadine	Antihistaminic	R06AX13	484	1,545	0.313
Meropenem	Beta-lactam antibiotic	J01DH02	2,586	8,424	0.307
Promethazine hydrochloride	Antihistaminic	R06AD02	1,843	6,163	0.299
Teicoplanin	Polypeptide antibiotic	J01XA02	558	1,933	0.289
Nystatin	Antifungal drug	A07AA02	1,827	6,572	0.278
Fusidic acid	Other antibiotics	J01XC01	650	2,914	0.223
Ceftizoxime sodium	Cephalosporins	J01DD07	1,738	8,574	0.203
Ceftazidime	Cephalosporins	J01DD02	1,056	5,309	0.199
Cefpiramide	Cephalosporins	J01DD05	620	3,636	0.168
Cefepime	Cephalosporins	J01DE01	406	2,443	0.166
Linezolid	Oxazolidinone antibiotics	J01XX08	630	4,169	0.151
Cefoperazone sodium and sulbactam sodium	Beta-lactam antibiotic	J01DD62	6,062	41,298	0.147
Milrinone	Cardiotonic drug	C01CE02	456	3,187	0.143
Heparin	Anticoagulant drug	B01AB06	2,883	23,439	0.123
Latamoxef sodium	Beta-lactam antibiotic	J01DD06	4,982	40,751	0.122

Abbreviations: DITP: Drug-induced thrombocytopenia; ATC: anatomical therapeutic chemical.

### Association of Suspected Drugs and Thrombocytopenia

The results of data extraction for the suspected drugs for each step are presented in Table 2. For detection of the DITP signals, the median number of patients enrolled in the drug exposure groups was 2,561 [interquartile range (IQR): 1,301–4,360] ranging from 314 (amphotericin B) to 14,122 (heparin), and the median number of patients enrolled in the comparison groups was 160,852 (IQR: 158,271–163,247.5) ranging from 142,067 (latamoxef sodium) to 164,685 (amphotericin B). The exposed and unexposed groups were matched according to the propensity score. The basic clinical information between two groups of each drug before and after PS matching is given in Supplementary Table S3, respectively.

**TABLE 2:** The data filtering workflow for suspected drugs.

Suspected drugs	Exposed group					Unexposed group				
	Exposed to suspected drug	At least 1 PLT test before and after medication	Initial PLT within LLN	Without thrombogenesis disease	Without thrombocytopenic agents	Not exposed to suspected drug	At least 2 PLT tests	Initial PLT tests within LLN	Without thrombogenesis disease	Without thrombocytopenic agents
Amphotericin	2,158	1,336	565	475	301	537,803	200,928	181,818	174,828	165,301
Chlorpheniramine	35,312	17,769	9,808	8,511	5,104	504,649	178,055	166,617	161,434	157,872
Vancomycin	19,883	10,209	5,072	4,591	3,354	520,078	187,900	172,756	166,530	160,309
Imipenem	4,089	2,134	929	888	738	535,872	199,242	181,071	174,103	164,753
Fluconazole	11,029	7,524	5,093	4,615	3,921	528,932	193,429	175,440	169,001	160,680
Sulfamethoxazole	51,402	8,629	6,987	6,205	5,025	488,559	164,747	152,583	148,508	146,549
Loratadine	2,214	901	300	775	543	201,023	181,476	13,902	174,476	164,855
Meropenem	14,555	7,492	4,366	4,050	3,517	525,406	191,224	174,328	167,794	159,488
Promethazine hydrochloride	11,669	5,946	4,126	3,736	2,953	528,292	194,872	176,672	170,036	161,499
Teicoplanin	3,207	1,841	1,239	1,147	960	536,754	200,094	180,912	173,919	164,309
Nystatin	11,554	6,157	4,385	3,817	3,000	528,407	193,292	175,231	168,789	160,354
Fusidic acid	4,356	2,622	1,956	1,751	1,597	535,605	199,048	179,798	172,920	163,295
Ceftizoxime sodium	14,348	4,778	3,337	2,974	2,368	525,613	192,543	174,294	167,826	159,204
Ceftazidime	10,207	3,349	2,370	2,217	1,838	529,754	196,103	177,401	170,493	161,410
Cefpiramide	5,600	2,308	1,804	1,593	1,445	534,361	198,562	179,191	172,391	162,744
Cefepime	3,333	1,734	1,438	1,363	1,266	536,628	200,028	180,472	173,427	163,696
Linezolid	5,646	2,776	2,119	2,051	1,866	534,315	198,635	179,609	172,558	162,927
Cefoperazone sodium and sulbactam sodium	28,166	12,811	10,662	10,257	9,456	511,795	181,827	164,766	158,245	149,853
Milrinone	3,762	2,929	2,708	2,705	2,570	536,199	199,543	179,811	172,646	162,799
Heparin	44,967	16,226	14,730	14,263	13,707	494,994	175,409	158,973	153,394	144,929
Latamoxef sodium	50,560	13,789	10,805	10,176	8,818	489,401	171,685	155,674	149,768	142,492

Abbreviations: PLT: platelet count LLN: lower limit of normal.

**FIGURE 2:**
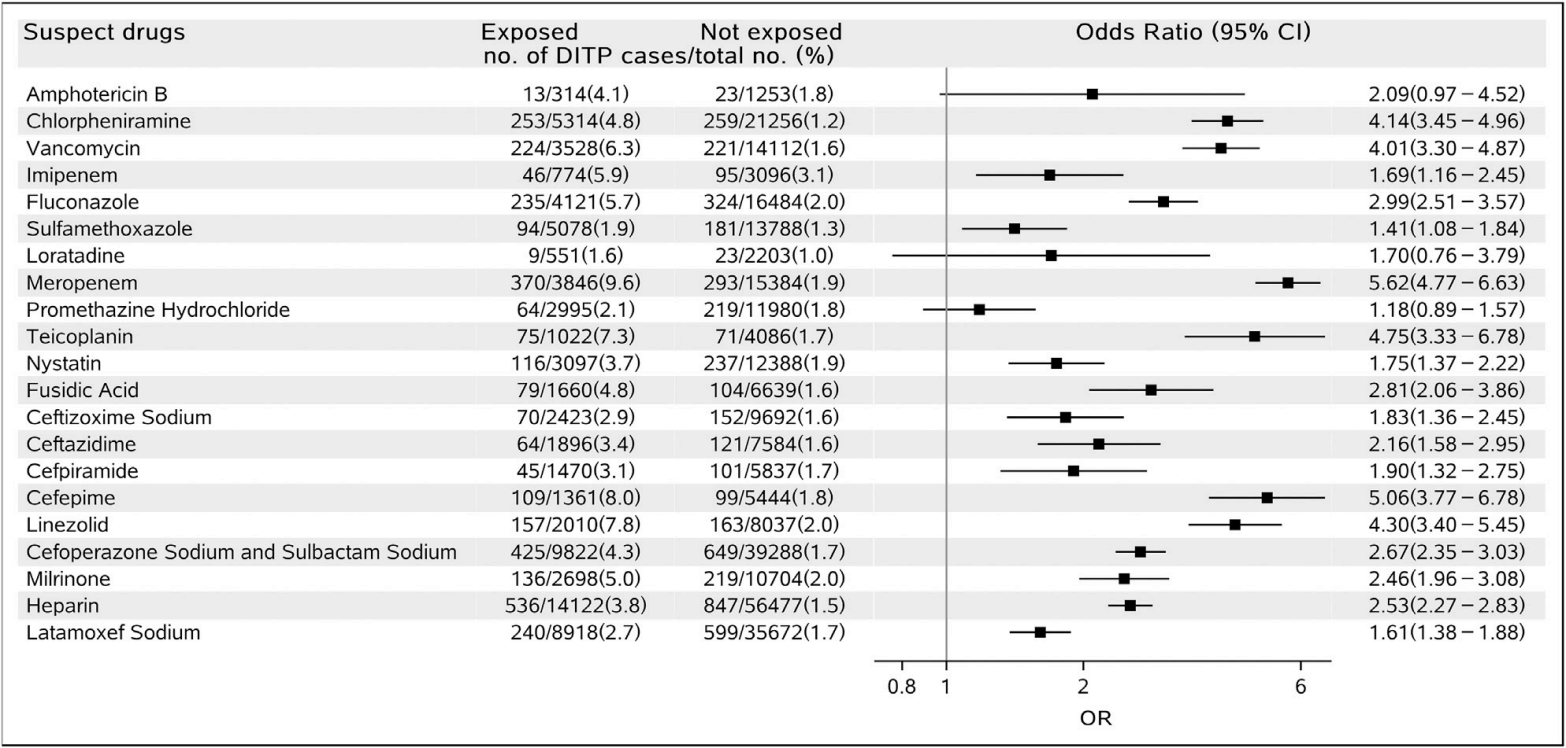
Forest plot for the association of suspected drugs and DITP.

Of the 21 suspected drugs, 18 showed positive signals including 14 anti-infective drugs (vancomycin, imipenem, fluconazole, sulfamethoxazole, meropenem, teicoplanin, fusidic acid, ceftizoxime sodium, ceftazidime, cefpiramide, cefepime, linezolid, cefoperazone sodium and sulbactam sodium, and latamoxef sodium; all OR>1, *p* < 0.001, see details in Figure 2), one antihistaminics (chlorpheniramine; OR: 4.14, 95% CI: 3.45–4.96, *p* < 0.001), one antifungal (nystatin; OR: 1.75, 95% CI: 1.37–2.22, *p* < 0.001), one cardiotonic (milrinone; OR: 2.45, 95% CI: 1.96–3.08, *p* < 0.001), and one anticoagulant (heparin; OR: 2.53, 95% CI: 2.27–2.83, *p* < 0.001). The remaining three drugs (amphotericin B, loratadine, and promethazine hydrochloride) were found not associated with DITP. The detailed results of all the 21 drug–DITP associations are shown in Figure 2.

Results from sensitivity analyses also showed similar results for each drug (18 drugs were potentially associated with DITP, and two drugs were not statistically associated with DITP), except for amphotericin B. The OR of amphotericin B and DITP was 2.24 (95% CI: 1.22, 3.76), which was different from that in the primary analysis using the PS matching method (Supplementary Table S4).

### Signal Novelty Evaluation

The novelty of 18 positive DITP signals observed in stage 2 was further evaluated through SPCs and current literature (Table 3). Two drugs, namely, nystatin and latamoxef sodium, were found to be possible new signals for Type I DITP as the adverse reactions had never been reported in SPCs and not been previously documented in the literature, both in children and adults. In addition, six drugs, namely, imipenem, teicoplanin, fusidic acid, ceftizoxime sodium, ceftazidime, and cefepime, were considered new signals for Type II DITP since they have not been found associated with thrombocytopenia in pediatric patients, although these associations have been reported in adults. The remaining ten drugs have been reported to be associated with thrombocytopenia in both adult and pediatric patients.

**TABLE 3:** The novelty of the positive signals of DITP.

Suspected drugs	Literatures of pubmed/Embase^a^		Literatures of Chinese database (CNKI/Wangfang)^a^		SPCs ^b^	Signal type*
	Adults	Children	Adults	Children		
Chlorpheniramine	√	×	√	√	×	known
Vancomycin	√	√	√	√	√	known
Imipenem	√	×	√	×	√	II
Fluconazole	√	√	√	√	√	known
Sulfamethoxazole	√	√	√	√	√	known
Meropenem	√	√	√	√	√	known
Teicoplanin	√	×	√	×	√	II
Nystatin	×	×	×	×	×	I
Fusidic acid	√	×	√	×	×	II
Ceftizoxime sodium	×	×	√	×	√	II
Ceftazidime	√	×	√	×	√	II
Cefpiramide	×	×	√	√	×	known
Cefepime	√	×	√	×	√	II
Linezolid	√	√	√	√	√	known
Cefoperazone sodium and sulbactam sodium	√	√	√	√	√	known
Milrinone	√	√	√	×	×	known
Heparin	√	√	√	√	√	known
Latamoxef sodium	×	×	×	×	×	I

Abbreviations: DITP: Drug-induced thrombocytopenia; SPCs: Summary of product characteristics.

a : Literature reviewed: 1) PUBMED:https://pubmed.ncbi.nlm.nih.gov; 2) Embase:https://www.embase.com; 3) Wanfang: http://www.wanfangdata.com.cn/index.html); 4) CNKI:https://www.cnki.net.

b : SPCs reviewed: 1) Micromedex: https://www.ibm.com/watson-health/learn/micromedex); 2) FDA website: https://www.fda.gov/; (3) Drug instructions.

* Signal type I: The specific drug-DITP signal had never been reported in the summary of product characteristics or in theliterature; II: the specific drug signal had been reported in the literature about adults, but no reports about children could be found in the literature; known: the specific drug-DITP association had been reported.

## Discussion

### Summary Results

Using the two-stage data-driven algorithm, our study found that nystatin and latamoxef sodium were associated with DITP in both adults and children. In addition, imipenem, teicoplanin, fusidic acid, ceftizoxime sodium, ceftazidime, and cefepime were associated with DITP in children. These drugs may be the suspected drugs for post-marketing surveillance and regulation.

George JN et al. systematically reviewed the published case reports about DITP from 1966 to 1997 (George et al., 1998) and established the DITP database based on the results both in adults and children. Then the authors updated this database regularly till 2018 (Rizvi et al., 1999; Arnold et al., 2013; Reese et al., 2013). Seven positive drug–DITP associations found in our study have been widely known in both adults and children, as confirmed by the DITP database involving both individual patient reports and group patient reports. Lisa M et al. assessed the performance of the clinical decision support system, which used an abnormal laboratory value alert rather than included the control group in detecting DITP in critically ill adult patients (Harinstein et al., 2012). According to this study, heparin, vancomycin, cefepime, and meropenem were associated with DITP alerts, which was in accordance with our findings.

Drug-induced thrombocytopenia has been associated with hundreds of medications and can lead to devastating consequences for the patient, especially for critically ill patients (Harinstein et al., 2012). Often the cause of DITP is not recognized in a timely manner, resulting in recurrent thrombocytopenia and inappropriate treatments. In addition, drug-dependent antibodies can persist for many years, and patients must be advised to avoid the drug that caused thrombocytopenia indefinitely (George and Aster, 2009). Greater publicity will increase awareness and suspicion of DITP among pediatricians and improve clinicians’ ability to evaluate, accurately diagnose, and manage patients who present with unexpected thrombocytopenia (Reese et al., 2013). Our study found eighteen positive signals of DITP, including six new signals in a pediatric population. When children are treated with such drugs, pediatricians should pay more attention and monitor the platelet count to prevent or minimize the risk of DITP in children. In addition, these signal drugs could be the candidate target drugs for further signal validation studies.

### New Signals of DITP

The association of nystatin with DITP was found to be a potential new signal in this study for the first time. Nystatin is an antifungal agent widely used to treat oropharyngeal candidiasis and cutaneous and mucocutaneous infections in pediatrics. The adverse effects listed in its SPCs include diarrhea, nausea, vomiting, abdominal pain, hypersensitivity reaction, and Stevens-Johnson syndrome. Nystatin, a class of tetraene macrolide antibiotics produced by *S. nunsei* and structurally similar to amphotericin B, is a kind of polyene macrolide antibiotic that mainly inhibits the cholesterol in the cell membranes of fungi and *mycoplasma*. Although we did not find any reports about nystatin-induced thrombocytopenia, the other polyene macrolide antifungal drug amphotericin B also acted on sterols of fungal cell membranes, which was documented to induce thrombocytopenia in some cases. One *in vitro* study found that amphotericin B’s effect on platelet membrane GP (GP1b) was concentration-dependent and could be influenced by duration of platelet storage (i.e., amphotericin B only affected platelets stored for 5 days versus fresh platelets) (Loo et al., 2012). Further investigations about the potential association between nystatin and thrombocytopenia are still needed.

The association between latamoxef sodium and thrombocytopenia was considered another new signal. Latamoxef sodium is a beta-lactam antibiotic used to treat various infections caused by sensitive bacteria. The mechanism of some other kinds of beta-lactam antibiotic–induced thrombocytopenia were that they could induce the production of antibodies, which would bind to platelet membrane protein only in the presence of drug or interact with platelet antigen (Yan et al., 2009; Loo et al., 2012). Despite no reports of latamoxef sodium–associated thrombocytopenia, our results were the first to show that latamoxef sodium might be associated with adverse thrombocytopenia in children. However, these findings will need further investigation to be confirmed.

Other six drug–DITP associations (imipenem, teicoplanin, fusidic acid, ceftizoxime sodium, ceftazidime, and cefepime) were identified as potentially new signals in children. All these drugs are anti-infectives for systematic use (their ATC codes are classified in J). Imipenem is a new class of carbapenem antibiotics, and it has the broadest antibacterial activity of all antibiotics available for systemic use in humans (Hellinger and Brewer, 1991). Some adult case reports were documented that imipenem/cilastatin induced acute thrombocytopenia (Alegre Herrera et al., 2001). However, there are only a few reports of thrombocytopenia associated with clinical doses of teicoplanin, a glycopeptide antibiotic used against Gram-positive bacteria (Zhang et al., 2014). The mechanism is based on the GPIIb/IIIa complex, which is a major target antigen of these teicoplanin-dependent antibodies (Garner et al., 2005). Fusidic acid is an active agent against a wide variety of Gram-positive bacteria, and it has been increasingly used in methicillin-resistant *Staphylococcus aureus* infection. The hematological side effects such as thrombocytopenia have been rarely reported in European and Asian adult populations (El-Kassar et al., 1996; Liao et al., 2003). Ceftizoxime sodium, ceftazidime, and cefepime are three cephalosporin antibiotics, of which the first two drugs are third-generation cephalosporins, and the last belongs to the fourth generation. A ceftazidime-induced thrombocytopenia case of an adult patient was reported for the first time in the Spanish pharmacovigilance system (Domingo-Chiva et al., 2017). Similarly, there is limited post-marketing surveillance evidence on thrombocytopenia associated with cefepime and ceftizoxime sodium (Lim et al., 2011). Because a delay in recognition can lead to significant morbidity and mortality, clinical criteria such as the Naranjo Adverse Drug Reaction Probability Scale were used to help determine the risk of DITP, which were less efficient. By contrast, our algorithm based on EMR data could be a referential experience to provide more clues for pediatric drug post-marketing pharmacovigilance.

### Strengths and Limitations of the Study

Compared with the proposed tool with those based on the spontaneous reporting system, our study integrated multisource data from the hospital information systems, biochemical laboratory, and drug prescription records. The active surveillance based on the routinely collected data integration is an effective approach for pharmacovigilance, which can detect a previously unrecognized adverse drug signal in the real practice immediately as well as provide more detailed information about symptoms, signs, diagnosis, timing sequence, and medication to analyze the potential association for drug–ADR pairs. Recently, some novel studies about ADR signal detection have been developed. Lee S et al. developed a comprehensive controlled vocabulary-based ADR signal dictionary and integrated this tool with an electronic health record for real-time large-scale pharmacovigilance studies (Lee et al., 2019). When detecting DITP signals through this integration tool, the controlled thrombocytopenia terms, including the Unified Medical Language System (UMLS) code, Logical Observation Identifiers Names and Codes (LOINC), standard nursing statement code, Medical Dictionary for Regulatory Activities (MedDRA) code, and ICD-10 code can be easily mapped. Most cases of DITP are caused by drug-dependent antibodies that are specific for the drug structure and bind tightly to platelets by their Fab regions but only in the presence of the drug (George and Aster, 2009). Yoon DY et al. established a series of electronic health record (EHR)–based pharmacovigilance methods called the BASE, CLEAR, and MetaLAB for laboratory abnormalities (Lee et al., 2017). Our study used a two-stage data-driven drug screening and PS matching method to detect children’s DITP signals. It is important to realize that this is a tool to assist with detection but does not ensure the identification of ADRs. In comparison with the CLEAR method, our two-stage designed approach has several advantages. In the process of selecting the drugs suspected to cause DITP, we assessed the potentialities by computing the crude incidence of ADEs to drug users. This crucial additional step increased the efficiency and speed of subsequent steps. In addition, more complicated confounders, such as relevant diagnoses with clear competing causes and medications that may affect the level of relevant laboratory indicators, were excluded to enhance the reliability and accuracy of the results. These results suggested that our method is a valuable tool to facilitate earlier signal detection using routinely collected EMR data.

Given that this study was a hospital-based observational design, several limitations on this research should be noted. First, although testing for antibiotic-induced antiplatelet antibodies remains the gold standard in the diagnosis of DITP, we had no access to the laboratory confirmation of DITP at the time of initial presentation because tests for drug-dependent antiplatelet antibodies are not available in most clinical laboratories. Given the feasibility, we chose PLT counts as the trigger of DITP to detect signals. Second, dose-related effects and possible residual confounders, such as concomitant drugs and the time-varying confounding by underlying diseases, were not controlled, leading to potential bias. Third, since our study is only based on EMR data from a single center, the sample size of some exposure to specific drugs, such as amphotericin B, was small and limited, which could lead to poor representation of results. Regulatory agencies have spared no effort for facilitating ADE signal detection through multiple heterogeneous data sources at present (Ali et al., 2020; Huang et al., 2021; Létinier et al., 2021).

Notable progress has been made in China in establishing the project named “China ADR Sentinel Surveillance Alliance” (CASSA) (Zhao et al., 2018). At present, we have developed an automated program based on this algorithm. Further, in the next step, more attention will be paid to integrate these multiple modules into a drug safety monitoring platform to support quick-response tools for pediatric clinicians and pharmacists in multicenter hospitals through a common data model (CDM), just like the Sentinel Initiative of FDA. Future research will also focus on tighter integration of the structured data and clinical narratives in EMR data to improve the accuracy and scalability of the method.

## Conclusion

In this study, we developed a pharmacovigilance method to explore potential DITP signals using routine EMR data. The two-stage designed algorithm was performed to first select suspected drugs and then determine the associations between DITP and drugs. Eighteen positive signals of DITP, including six new signals in children, were detected. Our study promotes the application of EMR datasets in pharmacovigilance and offers candidate drugs for further causality assessment studies.

## Data Availability

The original contributions presented in the study are included in the article/Supplementary Material; further inquiries can be directed to the corresponding authors.
