# Supplementary material for: Detection of Drug-Induced Thrombocytopenia Signals in Children Using Routine Electronic Medical Records
Source: Front Pharmacol. 2021 Nov 12;12:756207. doi: 10.3389/fphar.2021.756207 (PMC8633439; doi:10.3389/fphar.2021.756207)
Supplement: Supplementary file 1 [file DataSheet1.docx]

Supplementary Material

**Table S1. The excluded diseases with clear competing effects of thrombogenesis[1]**

| **Category** | **ICD-10 Codes** |
| --- | --- |
| Aplastic anemia | D61.905、D61.903、D61.801 、D61.101、D61.003、D61.001、D60.901、D59.501 |
| Autoimmune hemolytic anemia | D59.102、 D59.051、D59.251 |
| Autoimmune hepatitis | K75.809 |
| Bone marrow proliferation | C94.451、C94.051 |
| Chronic liver disease | E14.652、K71.051、K71.351、K71.552、K71.851、K76.901、 E05.903+ |
| Congenital thrombocytopenia | D69.404 |
| Desmosis | M35.101、M35.105、M35.901 |
| Disseminated intravascular coagulation | D65.X01、P60.X01 |
| Hemolytic uremic syndrome | D59.301 |
| Hypersplenism | R94.853 |
| Hypogamma globulin | D80.151、D80.051、D80.101 |
| Langerhans cell histiocytosis | D76.304 |
| Leucocythemia | C90.1、C91.0、C91.1、C91.2、C91.4、C92.-、C93.0、C93.1、C93.3、C93.7、C93.9、C94.2、C94.3 |
| Liver cirrhosis | K74.151、K74.251、K74.654 |
| Lymphoid hyperplasia | D82.351 |
| Lymphoma | C91.5、C96.3、C85.7、C84.5、C84.4、C84.3、C84.2、C82.7 |
| Myeloproliferative disorder | D46.901、D46.751 |
| Myelosuppression | D75.808、 D75.805、D61.902、D61.901 |
| Primary immune thrombocytopenia | D69.402 |
| Pseudothrombocytopenia | D69.601 |
| Rheumatoid arthritis | M05.301、M05.302、 M05.306+、M05.951、M06.001、M06.991、M08.001 |
| Scleroderma | L94.052、L94.151、M34.005、M34.801+、M34.901、M34.902 |
| Systemic lupus erythematosus | M32.006、M32.155+、M32.901、P00.852 |
| Thrombocytopenia | D69.602、D69.504、P61.001、D69.502 |
| Thrombocytopenic purpura | M31.101、P61.051、D69.406、D69.501、D69.553、D69.405、D69.351 |
| Thyroid disorder | A18.811+、C73、C79.808、F06.913、P72.051、P72.101、P72.151、P72.251、 R94.652、E05.901 |
| Vasculitis | I77.502、I77.605、I77.607、M31.003、D69.001 |

Abbreviations: ICD-10, international classification of diseases (Version 10).

**Table S2. The excluded drugs with effects of stimulate thrombogenesis [1]**

| **ATC** | **Drugs** | **Dosage form** |
| --- | --- | --- |
| L03AA02 | Filgrastim/granulocyte macrophage colony stimulating factor | Injection |
| H02AB04 | Methylprednisolone sodium succinate | Injection |
| L01XC02 | Rituximab | Injection |
| L03AA | Colony-stimulating factor Molgramostim/recombinant human granulocyte-macrophage | Injection |
| L03AA03 | colony-stimulating factor | Injection |
| D07AA01 | Methylprednisolone | Injection/Tablets |
| B05AX02 | Recombinant thrombopoietin | Injection |
| S01XA18 | Ciclosporin | Injection/Capsules/Oral solution |
| L04AA10 | Sirolimus | Oral solution |
| B02BX05 | Eltrombopag | Tablets |
| J06BA02 | Gamma globulin | Injection |
| D10AA03 | Dexamethasone | Injection/Tablets |

**Table S4. Sensitivity analyses of associations between suspect drugs-and thrombocytopenia using PS regression**

| **Suspect drugs** | **Exposed group** | | **Unexposed group** | | ***β*** | ***P* value** | **OR(95%CI)** |
| --- | --- | --- | --- | --- | --- | --- | --- |
|  | number of DITP events | number of non- DITP events | number of DITP events | number of non- DITP events |  |  |  |
| Amphotericin | 13 | 301 | 2662 | 162023 | 0.808 | 0.005 | 2.244(1.218,3.764) |
| Chlorpheniramine | 253 | 5061 | 2135 | 155137 | 1.318 | <0.001 | 3.735(3.236,4.294) |
| Vancomycin | 224 | 3304 | 2226 | 157507 | 1.456 | <0.001 | 4.290(3.710,4.936) |
| Imipenem | 46 | 728 | 2624 | 161517 | 0.908 | <0.001 | 2.479(1.803,3.324) |
| Fluconazole | 235 | 3886 | 2256 | 157830 | 1.222 | <0.001 | 3.395(2.944,3.897) |
| Sulfamethoxazole | 94 | 4984 | 2082 | 143866 | 0.522 | 0.008 | 1.168(1.017,1.472) |
| Loratadine | 9 | 542 | 2664 | 161578 | 0.115 | 0.733 | 1.122(0.535,2.046) |
| Meropenem | 370 | 3476 | 2086 | 156819 | 1.800 | <0.001 | 6.047(5.361,6.805) |
| Promethazine Hydrochloride | 64 | 2931 | 2580 | 158323 | 0.139 | 0.287 | 1.149(0.881,1.470) |
| Teicoplanin | 75 | 947 | 2568 | 161137 | 1.414 | <0.001 | 4.113(3.208,5.195) |
| Nystatin | 116 | 2981 | 2436 | 157315 | 0.673 | <0.001 | 1.961(1.611,2.363) |
| Fusidic Acid | 79 | 1581 | 2575 | 160118 | 1.048 | <0.001 | 2.851(2.240,3.577) |
| Ceftizoxime Sodium | 70 | 2353 | 2532 | 156072 | 0.685 | <0.001 | 1.983(1.542,2.506) |
| Ceftazidime Pentahydrate | 64 | 1832 | 2565 | 158236 | 0.710 | <0.001 | 2.033(1.560,2.603) |
| Cefpiramide | 45 | 1428 | 2619 | 159529 | 0.764 | <0.001 | 2.147(1.545,2.911) |
| Cefepime | 109 | 1252 | 2584 | 160511 | 1.462 | <0.001 | 4.313(3.501,5.259) |
| Linezolid | 157 | 1853 | 2537 | 159784 | 1.520 | <0.001 | 4.571(3.835,5.412) |
| Cefoperazone Sodium and Sulbactam Sodium | 425 | 9397 | 1915 | 147400 | 1.024 | <0.001 | 2.784(2.493,3.102) |
| Domperidone | 18 | 1102 | 2582 | 161209 | (0.075) | 0.753 | 0.928(0.559,1.435) |
| Milrinone | 136 | 2563 | 2391 | 159794 | 0.953 | <0.001 | 2.594(2.136,3.127) |
| Heparin | 536 | 13586 | 2049 | 142394 | 0.992 | <0.001 | 2.697(2.443,2.972) |
| Latamoxef Sodium | 240 | 8678 | 2162 | 139905 | 0.506 | <0.001 | 1.659(1.443,1.898) |

**Reference**

1. Reese JA, Li X, Hauben M, Aster RH, Bougie DW, Curtis BR, et al. Identifying drugs that cause acute thrombocytopenia: an analysis using 3 distinct methods. Blood 2010; 116:2127-2133.
